# The Characteristics of Dialysis Membranes: Benefits of the AN69 Membrane in Hemodialysis Patients

**DOI:** 10.3390/jcm12031123

**Published:** 2023-01-31

**Authors:** Shuzo Kobayashi, Takayasu Ohtake

**Affiliations:** Kidney Disease and Transplant Center, Shonan Kamakura General Hospital, 1370-1 Okamoto, Kamakura-City 247-8533, Kanagawa, Japan

**Keywords:** hemodialysis, dialysis membrane, AN69, PAD, LDL-apheresis

## Abstract

Patients undergoing hemodialysis (HD) experience serious cardiovascular complications, through malnutrition, inflammation, and atherosclerosis. Amputation for peripheral arterial disease (PAD) is more prevalent in patients undergoing HD than in the general population. In addition, revascularization procedures in dialysis patients are often associated with subsequent amputation and high mortality rates. To improve the prognosis of dialysis patients, malnutrition and inflammation must be properly treated, which necessitates a better understanding of the characteristics of dialysis membranes. Herein, the characteristics of several dialysis membranes were studied, with a special reference to the AN69 membrane, noting several similarities to low-density lipoprotein (LDL)-apheresis, which is also applicable for the treatment of PAD. Both systems (LDL-apheresis and AN69) have anti-inflammatory and anti-thrombogenic effects because they use a negatively charged surface for extracorporeal adsorptive filtration from the blood/plasma, and contact phase activation. The concomitant use of both these therapeutic systems may have additive therapeutic benefits in HD patients. Here, we reviewed the characteristics of dialysis membranes and benefits of the AN69 membrane in dialysis patients.

## 1. Burden of Chronic Hemodialysis (HD) in Japan: Epidemiological and Economic Perspectives

Japan is estimated to have the highest number of dialysis patients, and this number continues to increase every year [1]. Estimates from the Japanese Society for Dialysis Therapy indicate that currently, one out of 385.1 Japanese citizens are dialysis patients. The number of chronic dialysis patients per million in 2016 had increased to 2596.7 from 2557.0 in 2015, and at the end of 2016, 76,836 patients were undergoing hemodiafiltration (HDF) and 635 patients were treated by home hemodialysis (HD) therapy, indicating an increase of 63 patients from 2015 [2]. At the end of 2017, the number of chronic dialysis patients had reached 334,505, an increase of 4896 patients from 2016 [2]. However, the number of peritoneal dialysis (PD) patients has gradually decreased since 2014, the number of PD patients in 2015 and 2016 being 9322 and 9021, respectively. Again, 20.3% of the PD patients were on combination therapy with either HD or HDF therapy [1]. Japan is also plagued by an increase in the proportion of elderly patients (70 years and above) who remain on dialysis [3]. The financial burden of renal diseases is particularly high, with the average medical cost being 14.5 times higher in individuals with renal disease than in those without renal disease/dialysis. In Japan, the estimated medical costs incurred for treating renal diseases were approximately 1.546 trillion yen in 2016, accounting for 3.8% of the total healthcare expenditure that year [4]. In Japan, in-center dialysis, home dialysis, and transplantation are the available options for the treatment of end-stage renal disease (ESRD); however, the use of transplantation and home dialysis is generally very low [4].

## 2. Risk Factors and Complications Associated with Chronic HD

In Japan, HD is considered as a mainstream renal replacement therapy and used in 95% of patients suffering from chronic kidney disease (CKD) [5]. The most common reasons for dialysis use in Japanese patients include diabetic nephropathy (38.8%), chronic glomerulonephritis (28.8%), and nephrosclerosis (9.9%) [2]. Diabetes mellitus is a well-known risk factor for CKD. Recent estimates indicate that the cumulative survival of chronic HD patients with poor glycemic control is significantly lower than that of patients with fair or good glycemic control [6].

Cardiovascular disease (CVD) is the main cause of mortality in dialysis patients [7]. The increased cardiovascular risk in CKD patients may be attributed to hypertension that may occur due to the activation of the renin–angiotensin–aldosterone system, vascular calcification associated with abnormal metabolism of calcium and phosphorus, and the specific dyslipidemia of CKD, chronic inflammation, malnutrition, oxidative stress, and uremic factors [7,8]. 

Observational studies in Japanese dialysis patients have demonstrated a close relationship between dyslipidemia (hyper-low-density lipoprotein (LDL) cholesterolemia, hypo-high-density lipoprotein (HDL) cholesterolemia, hypertriglyceridemia, and/or hyper-non-HDL-cholesterolemia), the severity of atherosclerosis, and the risk of myocardial infarction. ‘The Japanese Society for Dialysis Therapy Guidelines for Management of Cardiovascular Diseases in Patients on Chronic Hemodialysis’ suggest that dyslipidemia is an independent risk factor for CVD, as it is closely associated with atherosclerosis, CVD, and myocardial infarction [9]. 

Hypertension in HD patients plays an important role in the development of CVD. Because of the variability of blood pressure within a week, weekly averaged blood pressure (WAB) is a useful prognostic marker for evaluating hypertension in HD patients [10].

Peripheral arterial disease (PAD), defined as obstructive atherosclerosis of the lower extremities, is associated with an increased risk of cardiovascular events, and an increased mortality rate in HD patients [11]. Moreover, PAD is characterized by a high morbidity rate in dialysis patients related to vascular calcification and a high mortality rate related to lower limb amputation. Vascular calcification is induced by PAD, and is difficult to treat [12,13]. Vascular calcification reportedly increases with a decline of glomerular filtration ratio (GFR) [14]. Both the prevalence and severity of PAD in HD patients are closely associated with arterial calcification in the lower limbs [15]. Arterial abnormalities may be caused by rheological abnormalities [16]. Compared to those in healthy individuals, leukocyte aggregates are increased in HD patients. Increased platelet/leukocyte aggregates are associated with atherosclerosis in these patients [16].

Moreover, patients undergoing dialysis often complain of uncomfortable symptoms such as pruritus, irritability, depression, insomnia, and intradialytic hypotension. The common pathogenesis of these dialysis-related complications could be explained by the uremic retention of solutes and bio-incompatibility of dialysis therapy, which may in turn lead to microinflammation [17]. With respect to these complications, malnutrition, inflammation, and atherosclerosis are the most important aspects to consider, as they cause the highest morbidity and mortality among dialysis patients [18]. In subsequent sections, we will discuss in detail the various dialysis-related complications and their clinical impact on patients undergoing chronic HD.

## 3. Malnutrition, Inflammation, and Atherosclerosis in HD: The Heart of the Matter

**Figure 1:**
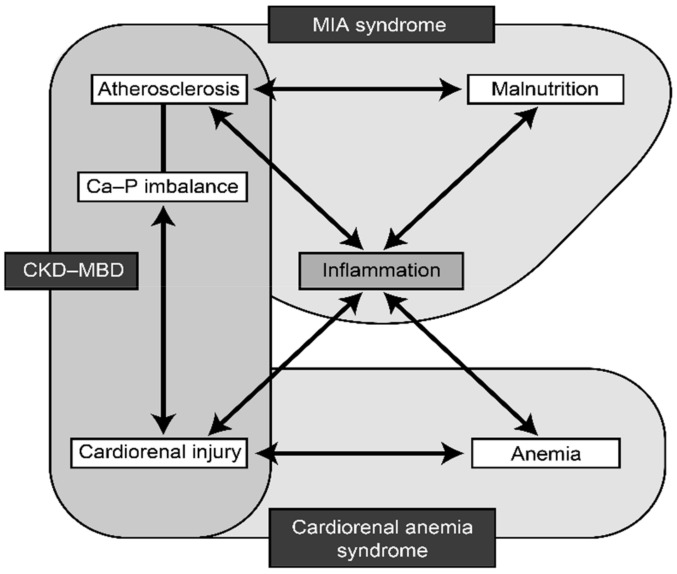
Interaction between malnutrition, inflammation, and atherosclerosis in chronic kidney disease [22]. MIA: malnutrition–inflammation–atherosclerosis; Ca: Calcium; P: Phosphorus; CKD-MBD: chronic kidney diseases-related mineral and bone disorder.

Malnutrition–inflammation–atherosclerosis (MIA) syndrome, CKD-related mineral and bone disorder (CKD-MBD), and cardio-renal-anemia (CRA) syndrome interact with each other in the setting of renal disease. MIA syndrome is a complex of microinflammation, malnutrition, and atherosclerosis, which mutually interact and form a vicious cycle leading to advanced atherosclerosis in dialysis patients [19]. CRA syndrome is a vicious complex of cardiovascular disease, renal failure, and anemia, which also form a vicious circle, with each one capable of causing or worsening of other components [20]. CKD-MBD includes abnormal mineral metabolism, deranged bone turnover and mineralization, and widespread distribution of ectopic calcification in soft tissues, including vascular calcification (medial calcification). Fibroblast growth factor-23 (FGF23) and its co-receptor, Klotho, are thought to have a central role in atherosclerosis and CKD-MBD [21]. Inflammation plays a central role in all the three conditions (Figure 1) [22].

## 4. Malnutrition and HD: Critical Connection

Malnutrition is common in HD patients and is a powerful predictor of morbidity and mortality. In chronic HD patients, low concentrations of serum albumin, blood urea nitrogen (BUN), serum creatinine, and low relative body weight are significantly associated with an increased risk of mortality. Malnutrition has been reported in 23–76% of HD patients and is dependent on factors such as the quality of dialysis therapy, case mix, comorbid conditions, and age [23]. Anorexia, hormonal and metabolic derangements, decreased nutrient intake, and catabolic factors are associated with the dialysis procedure selected. Additionally, the structure of the dialysis membrane plays an important role in dialysis-related malnutrition [24].

The elderly population undergoing dialysis has distinct characteristic features: malnutrition, protein-energy wasting (PEW), frailty, and sarcopenia [25]. A study conducted by Yasui et al. indicated that 15% of Japanese patients on HD have PEW. These findings further suggest that reduced muscle mass, lack of exercise, chronic inflammation, unintentional low dietary energy intake, and insulin resistance are the major contributing factors for PEW [26]. The optimum protein and calorie intake does not effectively help combat malnutrition in chronic HD patients. This is because multifactorial derangements cause malnutrition in chronic HD patients rather than poor nutritional intake alone [27].

## 5. Inflammation and HD

**Figure 2:**
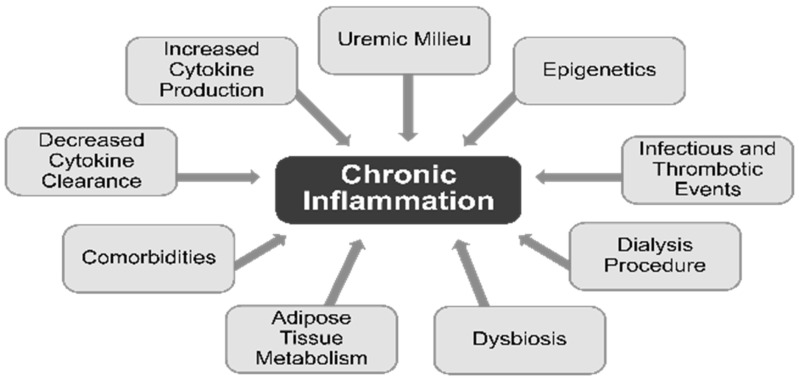
Inflammation in dialysis patients [28].

Numerous complications of chronic dialysis are attributed to inflammation, via monocyte release of interleukin (IL)-1, the master cytokine of inflammation. The development of inflammation in CKD begins well before the need for chronic dialysis. Elevated levels of inflammatory biomarkers such as IL-6 and C-reactive protein (CRP) suggest that CKD and chronic dialysis can both be regarded as low-grade inflammatory processes [28]. Multiple factors contribute to the chronic inflammatory activation in patients undergoing dialysis. The contributing factors for higher levels of circulating cytokines include decreased cytokine clearance and increased production of the cytokines, uremic milieu, epigenetic influences, infectious and thrombotic events, dialysis procedure, dysbiosis, adipose tissue metabolism, and comorbid conditions (Figure 2) [28]. Procedure-related factors that cause inflammation in dialysis patients include the use of nonsterile dialysate or non-biocompatible membranes and back leakage of dialysate across membranes [29].

Increased levels of inflammatory markers are associated with adverse clinical outcomes, including all-cause mortality, cardiovascular events, kidney disease progression, PEW, diminished motor function, and cognitive impairment. Other adverse consequences of elevated inflammatory markers include CKD-MBD, anemia, and insulin resistance [28].

## 6. Atherosclerosis in HD: PAD-Associated Heightened Risk of Cardiovascular Events

The risk of atherosclerosis is high in patients undergoing HD, which can lead to the development of PAD [30,31]. PAD is defined as obstructive atherosclerosis of the lower extremities and is associated with an increased risk of cardiovascular events and an increased mortality rate in HD patients [11]. A recent study in Japan reported a high prevalence rate of PAD in HD patients [30,31]. Several factors that are unique to dialysis patients may predispose them to the development of PAD, including manifestations of kidney disease, such as hyperparathyroidism, chronic inflammation, and hyperphosphatemia [11]. Because of its progressive nature, screening and diagnosis of PAD in dialysis patients are of utmost importance. Furthermore, neglecting PAD could lead to an increased risk of cardiovascular events and even amputation [11]. The ankle–brachial blood pressure index (ABI) is used as the standard tool to detect PAD. However, due to the presence of vascular calcification, ABI yields false-negative results. Therefore, skin perfusion pressure (SPP) is a more useful tool for detecting PAD in HD patients, with 84.9% accuracy [32]. 

Among non-dialysis patients with PAD, 1% to 3% with claudication underwent amputation within five years. Amputation for PAD is more prevalent in patients with ESRD than in the general population. In addition, revascularization procedures among dialysis patients are often associated with subsequent amputation and high mortality at the end of one year [11].

A high incidence of major amputation or death is a major clinical problem in HD patients. In chronic HD patients, critical limb ischemia (CLI) from isolated infra-popliteal artery disease is frequently observed and is usually treated with endovascular therapy (EVT) and lower extremity bypass surgery [33]. In a study by Nakano et al., the amputation-free survival (AFS) rates after EVT and bypass surgery were 66% and 61%, respectively, at 1 year [34]. However, an important limitation is that the angiographic restenosis rate is extremely high in patients with CLI after EVT or bypass surgery [35]. The study revealed that within a year after EVT, 70% of the patients with PAD underwent revascularization.

## 7. Physicochemical Structures and Key Features of Different Dialysis Membranes

### Different Types of Dialysis Membranes and Their Features

To reduce cardiovascular events, of importance to note is choosing an appropriate dialysis membrane. Dialysis membrane materials can be classified into three groups: unsubstituted cellulose, substituted (modified) cellulose, and synthetic [36,37,38]. Initially, unsubstituted cellulose membranes of large thickness and small pore size were used for HD. These membranes are inefficient for small solute removal and have side effects such as elevated levels of complement activation. Regenerated cellulose membranes were later developed via chemical modification to improve their biocompatibility by replacing their hydroxyl groups with acetate groups, such as cellulose acetate (CA), cellulose diacetate (CDA), or cellulose triacetate (CTA). Other membranes developed from regenerated cellulose have relatively better performance, including diethylaminoethyl (DEAE)-substituted cellulose and multilayer vitamin E-coated cellulose [39]. Subsequently, several types of synthetic membranes have been developed from different materials, such as polysulfone (PSu) [40], polyamide, polymethyl methacrylate, and polyacrylonitrile (PAN). Atherosclerosis, commonly observed in HD patients, is associated with an increase in leukocyte/platelet aggregates. Arterial abnormalities also result from rheological anomalies in the blood. In this context, vitamin E-coated hemodialyzers improve atherosclerotic changes in HD patients through positive effects on rheology in addition to antioxidant effects. It also helps to reduce the required erythropoietin (EPO) dose during HD [39].

The different types of dialysis membranes and their key features are listed in Table 1.

## 8. Impact of Dialysis Membranes on Clinical Outcomes

Reports indicate that the clinical outcomes of chronic HD patients are influenced by the type of dialyzer membrane used. This is particularly dependent on the solute mass transport efficiency and bio(in)compatibility of the dialyzer membrane [41,42].

The solute mass transport is mostly represented by the flux characteristics of the membrane and is expressed as the ultrafiltration (UF) coefficient in mL/mmHg/h. This represents the water permeability of the membrane [41,42].

Various reactions may be triggered by the contact of blood with the artificial surface of an inadequately biocompatible dialysis system. These interactions include leukocyte, complement, and thrombocyte activation, coagulation, and production of cytokines, free-oxygen radicals, β2-microglobulin, bradykinin, and other events. Reactions caused by inadequate biocompatibility may injure the patient. Hence, biocompatibility is considered one of the leading areas of concern in dialysis treatment. Dialysis membranes that cause minimal activation of plasma proteins or cellular cascades can be considered biocompatible.

## 9. Unique Biocompatibility and Selective Adsorptive Properties of the AN69 Membrane

### Introduction and History of the AN69 Membrane

The development of a synthetic membrane for use in dialysis was initiated in 1969 by a company named Rhône-Poulenc, following a request from the French government. This led to the development of the AN69 membrane. A copolymer of sodium methallyl sulfonate and acrylonitrile was used to manufacture an AN69 membrane. The unique feature of the AN69 membrane is that it is hydrophilic in nature compared with other synthetic membranes. This is because of the presence of sulfonate groups that create a hydrogel structure by attracting water, thereby providing hydraulic permeability with highly diffusive properties [43]. The AN69 membrane was first produced as a flat sheet; however, since 1980, it has been developed as a hollow fiber. The AN69 membrane has evolved continuously since its development in the early 1970s to meet the challenges and requirements of dialysis therapy. Its continuous advancement in thickness and internal diameter has led to improved performance. In the 1980s, the dialyzer manufacturing process was modified to allow sterilization by γ-radiation, instead of ethylene oxide [43]. The use of the AN69 membrane has been found to be associated with improved efficiency, reduced treatment duration, reduced risk of peripheral neuropathy, and improved clinical outcomes and quality of life. This paved the way for the initiation of volume-controlled, high-flux dialysis [43].

## 10. Key Features of the AN69 Membrane

### Adsorptive Features

The microstructure and chemical composition of the AN69 membrane facilitate the bulk absorption of low-molecular-weight proteins, such as basic proteins and inflammatory mediators. The adsorptive property of the AN69 membrane, specifically for basic medium-sized proteins, distinguishes it from other adsorptive membranes and synthetic high-flux dialysis membranes [43]. A study demonstrated that low-molecular-weight acidic proteins can be eliminated by filtration on negatively charged membranes (such as AN69) or uncharged membranes. Conversely, basic low-molecular-weight proteins can be removed by specific ionic interactions on the AN69 membrane [44]. The superior biocompatibility of the AN69 membrane is due to its unique adsorptive capacity for anaphylatoxin and inflammatory complement factors [43].

The hallmark features of the AN69 membrane are its high permeability to fluids, including a broad range of uremic retention products, and its excellent biocompatibility, measured using either novel or conventional indicators [43]. 

## 11. Effect on Inflammatory Response

During HD, exposure of the blood to foreign surfaces activates various defense mechanisms, including coagulation, fibrinolysis, and complement activation, via an alternative pathway. In turn, complement activation leads to impairment of the host defense, as a result of increased consumption of complement proteins [45]. It has been observed that the intensity of complement activation varies with the type of membrane used: for example, with cellulose (CU), a much more marked activation is observed when compared to synthetic PAN membrane [46]. 

Several studies have demonstrated that the AN69 membrane has a lower ability to activate the complement system because of its adsorptive properties when compared to other membranes, such as CA dialyzers and CU membranes [46,47,48,49].

Adverse effects of dialysis, such as fever, hypotension, and acute-phase inflammatory reactions, are linked to the production of activated monocytes and macrophages. These were IL-1, tumor necrosis factor (TNF)-α, and IL-6. Compared to the other membranes such as CU, the AN69 membrane neither induces cytokine production nor causes the activation of mononuclear cells [50,51]. Moreover, no changes in neutrophil and monocyte counts occur during HD with the AN69 membrane, unlike with the CU membrane [52]. 

Since high-flux dialysis membranes, such as the AN69 membrane, are highly permeable, concerns regarding their potential to permit the passage of cytokine-inducing residues across these membranes, either through back-diffusion or back-filtration, have been raised. However, in vitro studies have shown that the AN69 membrane is not permeable to specific types of bacterial endotoxins compared with the permeability of other membranes [53,54]. 

Recent advances in the manufacturing technique of dialysis membranes enabled the development of a new hemofilter with an AN69 surface-treated membrane (Oxiris) [55]. It provides high absorbance of endotoxin (negatively charged) and cytokines and excellent anti-thrombogenicity because of its positively charged surface [56,57]. Case series and studies have reported the hemofilter’s validity in reducing cytokine concentrations in COVID-19 patients [58,59,60]. 

## 12. Effect on Oxidative Stress and Carbonyl Stress

In addition to increased inflammation, HD is often associated with oxidative stress due to the activation of white blood cells, which triggers the generation of reactive oxygen species (ROS) and the loss of antioxidants during dialysis. Oxidative stress increases the risk of morbidity and mortality in this patient population and could be measured as advanced oxidation protein products in the plasma of uremic patients [61,62]. 

Evidence indicates that the AN69 membrane provides more protection from oxidative stress in HD patients than other membranes such as CDA [63]. Carbonyl stress is also implicated in long-term complications, such as atherosclerosis or dialysis-related amyloidosis, in ESRD patients [64,65]. Increased levels of advanced glycation end products (AGEs), which contribute to uremic toxicity, result from the accumulation of carbonyl AGE precursors in uremic plasma [66]. The effect of the AN69 membrane on carbonyl stress marker levels was similar to those of other membranes in a single HD session. However, in patients who were switched from PSu to the AN69 membrane, the carbonyl stress marker levels reduced to the control level [66].

## 13. Hemocompatibility

A high fibrinogen concentration is associated with increased cardiovascular risk and accelerated atherosclerosis. The AN69 membrane has good hemocompatibility, as it induces a lower thrombotic response, and fibrinogen and erythrocyte sedimentation rates are higher in non-biocompatible membranes [66].

## 14. Negative Charge

During the 1990s, the incidence of hypersensitivity reactions in HD patients, especially in those using electronegatively charged PAN membranes (AN69), increased significantly. This was due to the widespread use of antihypertensive drugs, such as angiotensin-I-converting enzyme inhibitors (ACEi) [67]. These inhibitors prevented the normal breakdown of bradykinin, the chief mediator of hypersensitivity reactions that occur during HD [68,69]. Similar reactions have also been reported with the use of PSu and other synthetic membranes during dialysis. The Evaluation of the Losartan in Hemodialysis (ELHE) study, which assessed the efficacy of an alternative antihypertensive drug, losartan, for HD patients, indicated a lower prevalence of anaphylactoid reactions compared to the use of ACEi when used in combination with the AN69 membrane [70]. To neutralize the electronegativity of the AN69 membrane and lower the generation of kinins, a membrane was developed with a coating of polyethyleneimine, called the AN69-ST (ST for surface treated) membrane [43]. They demonstrated lower adsorption of high-molecular–weight kininogen and contact-phase activation than the regular AN69 membrane [71].

## 15. Functional Similarities of AN69 with LDL-Apheresis

LDL-Apheresis is the process of removing LDLs from the plasma and was originally used for familial hyperlipidemia patients. Recommendations for initiation of LDL-apheresis in patients affected by hypercholesterolemia are controvercial, as no study demonstrated definitively improved survival with LDL-apheresis. International guidelines and systematic review recommend to consider LDL-apheresis in homozygotes or those with analogous phenotypes if the patient has already been treated with diet and pharmacotherapy and LDL cholesterol levels still remain higher than cut-off values based on age and cardiovascular state [72,73].

There are several methods to remove LDL cholesterol from the blood. These include heparin-induced extracorpoeral LDL cholesterol precipitation, immunoadsorption, double filtration plasma pheresis of lipoproteins, and liposorber system. Through selective adsorption, liposorber system LDL-apheresis removes LDL from plasma using negatively charged dextran beads [74]. In addition to the lipid-lowering function, several other beneficial effects of LDL-apheresis have been reported, including anti-inflammatory, anti-atherogenic, and anti-thrombotic effects [74,75]. Owing to its pleotropic benefits, LDL-apheresis is effective against PAD in HD patients, through the reduction of LDL, coagulation factors, and ROS production [76]. In this context, it is important to note that LDL-apheresis has several functional similarities with the AN69 membrane. Both of these systems use a negatively charged surface for extracorporeal adsorptive filtration from the blood/plasma, and contact phase activation has been associated with both these systems [77]. Similar to AN69, LDL-apheresis therapy leads to a reduced generation of cytokines and CRP and improved macrophage function, thereby eliciting its anti-inflammatory role. Similar to the protective role of AN69 in oxidative stress, LDL-apheresis lowers the ROS generation by leukocytes. As observed in the case of AN69, LDL-apheresis also improves hemorheology by increasing blood viscosity and lowering coagulant and fibrinogen levels [74]. 

In addition to the beneficial effects of AN69, it is associated with fewer complications in HD patients, even those with PAD, as compared to those associated with other common membranes [78]. LDL-apheresis has also been successfully used in HD patients with complications such as PAD, owing to its pleitropic benefits other than lipid-lowering effects [74,78]. Therefore, the concomitant use of both these therapeutic systems in specific patients, such as those with PAD, may provide additive therapeutic benefits in such HD patients.

## 16. Clinical Evidence of AN69 Membrane Use in Chronic HD Patients

The AN69 membrane is one such membrane that has favorable effects on dialysis because of its well-balanced removal of low-molecular–weight proteins and small solutes [10]. In this section, we will discuss the clinical evidence highlighting the benefits of AN69 membrane use in patients undergoing HD. Table 2 lists the different clinical studies evaluating the different effects of the AN69 membrane in the chronic HD setting.

## 17. Effect on Solute Removal, Hemodynamic Parameters, and Nutritional Status

**Figure 3:**
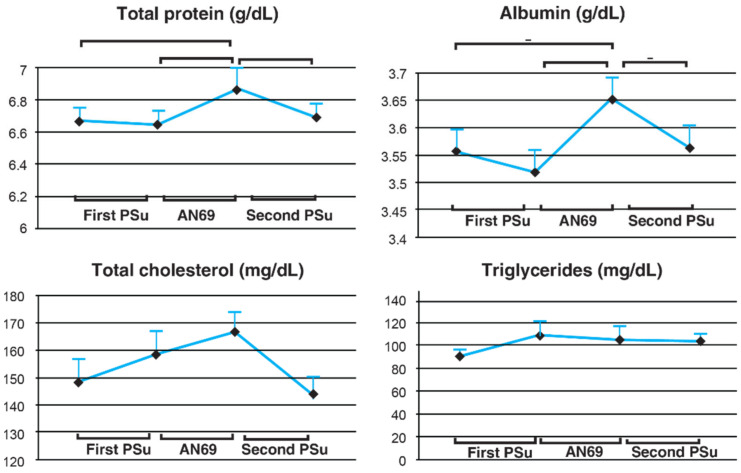
Changes in the nutritional indices during the PSu and AN69 periods [78]. PSu: polysulfone, AN69: acrylonitrile 69.

A crossover study conducted by Furuta et al. [78] among 28 elderly maintenance HD (MHD) patients aged 75 years or older compared AN69 with PSu membrane with respect to solute removal during HD, hemodynamic condition, and nutritional status after three months of treatment. In the last session of the first PSu period and the AN69 period, pre-and post-serum levels of IL-6 were measured and calculated for reduction ratio. At the start of the study and the last session of each membrane period, pre-HD serum total protein, albumin, total cholesterol, triglyceride levels were measured to compare the nutritional status among each treatment period. The study findings indicated that the reduction ratio for the inflammatory cytokine IL-6 was significantly higher for AN69 compared to PSu membrane (*p* < 0.05). This could be attributed to the negative charge present on the AN69 membrane surface that facilitates adsorption of various inflammatory cytokines such as IL-10, IL-6, and IL-18. The study findings also indicated that after three months of AN69 use, serum albumin, total protein, and cholesterol levels increased significantly and returned to baseline after switching back to PSu (Figure 3). Furthermore, the frequency of saline use to treat hypotension episodes decreased significantly during HD with AN69. The study revealed that, in elderly MHD patients, AN69 use led to improvements in both chronic inflammatory conditions and malnutrition. Therefore, for elderly HD patients, AN69 may be the preferred membrane for dialysis.

## 18. Effects on Solute Removal and Nutritional Status of Older HD Patients with Mild PAD 

### 18.1. Crossover Trial

A crossover trial [79] comparing the solute removal properties of AN69 and PSu membranes was conducted among six elderly patients with mild PAD (mean age: 70.8 ± 9.0 years) with stable hemodynamics and no detectable anemia; the patients were administered four-hour HD thrice a week; dialyzers were switched every two weeks, and parameters such as reduction rate, clearance, clear space, and amount of low-molecular-weight protein, β2-microglobulin, and low-molecular-weight solutes including creatinine, urea nitrogen, and inorganic phosphorus were evaluated. The reduction rate and removal amount of amino acids and albumin were also determined. Although AN69 was less efficient than PSu in the removal of β2-microglobulin and creatinine, the overall dialysis efficiency for the removal of low-molecular-weight solutes was similar for both membranes. Albumin leakage and amino acid removal were significantly lower in the case of AN69 than in PSu. The study concluded that, owing to the negative charge and pore size, albumin removal by AN69 was significantly lower than that of the other membranes, and that the use of AN69 may ameliorate the deterioration of symptoms in HD patients.

### 18.2. Long-Term Benefits

A study included eight elderly patients (mean age: 72.1 ± 10.6 years) who were switched from PSu membrane to AN69 and observed for 72 weeks to determine the long-term effects of AN69 use. Analyses for nutritional benefits and long-term effects included measurements of albumin levels and various parameters from blood, such as β2-microglobulin, CRP, low-density lipoprotein (LDL), fibrinogen, nitrogen oxide, hemoglobin, ferritin, and renal anemia. Both serum albumin and the geriatric nutritional risk index (GNRI) were maintained at stable levels. The GNRI level was maintained above 92, which is the target level for HD patients. All the parameters studied to assess the nutritional status, such as normalized protein catabolic rate, dry weight, and creatinine generation rate, remained stable throughout long-term use of the AN69 membrane. CRP and LDL levels are associated with the development of atherosclerosis. Although the patients had atherosclerosis, AN69 did not significantly alter the levels of CRP and LDL. No significant changes occurred in β2-microglobulin levels. The hemoglobin levels remained favorable and stable. Overall, in elderly HD patients with mild PAD, AN69 demonstrated good biocompatibility and HD efficiency [79]. 

## 19. Effect on Amino-Acid Loss into Dialysate during HD

Another study [80] conducted in Japan among nine maintenance HD patients evaluated the amino acid losses using three types of membranes: hydrophilic and nonhydrophilic polyester–polymer alloy membranes and the AN69 membrane. In the same order, patients received treatments with all three membranes at one-month intervals, without membrane reuse. Standard HD was administered three times a week for 3 to 4 h. Significant differences in the losses of tryptophan, cystine, phenylalanine, and ornithine were observed between the HD membranes (Table 3). The total amino acid loss was 72.1 ± 22.5 mg/L for the AN69 membrane, and 83.3 ± 16.1 mg/L and 85.7 ± 27.2 mg/L for nonhydrophilic and hydrophilic polyester–polymer alloy membranes, respectively [80].

Therefore, compared to polyester–polymer alloy membranes, AN69 leads to lower amino acid loss, thereby implying a better nutritional state with its use in maintenance HD (MHD) patients.

## 20. Other Benefits of the AN69 Membrane in HD Patients

### 20.1. Effect on Serum Hepcidin Levels

CKD patients have a dysregulated iron metabolism, leading to anemia of chronic disease (ACD). Liver hormone hepcidin controls iron homeostasis. Hepcidin is a negative regulator of intestinal iron absorption and iron release from macrophages. Hepcidin induces degradation of the iron exporter ferroportin to reduce iron entry into plasma from dietary sources and body stores. Iron deficiency and erythropoietic drive suppress hepcidin production to provide adequate iron for erythropoiesis [84]. Hepcidin excess, as a consequence of inflammation, decreased renal clearance, and reduced erythropoietin production, is suspected to cause the dysregulation of iron metabolism, resulting in ACD. 

#### 20.1.1. Ex Vivo Study

An ex vivo study [81] was performed using 50 mL of whole blood collected from healthy volunteers circulated for 2 h in a microcircuit with mini-dialyzers (acrylonitrile-co-methallyl sulfonate [AN69] or PSu without ultrafiltration). The levels of hepcidin-25 were measured in the blood samples at 0, 60, and 120 min. The study demonstrated that although serum hepcidin 25 levels increased after the ex vivo session with PS, they significantly decreased with AN69 after one and two hours (mean change ratio: −68 ± 39%). 

#### 20.1.2. In Vivo Study

An in vivo study included the collection of blood samples with 28 MHD patients at the start and end of HD sessions with the PS or AN69 membrane. The serum levels of hepcidin 20, 22, and 25 were measured using liquid chromatography tandem mass spectrometry. The serum levels of urea nitrogen and β2-microglobulin were also measured. The study findings indicated that the reduction of β2-microglobulin was significantly higher for PSu (62.4 ± 6.5%) than for the AN69 membrane (29.2 ± 8.2%). However, the reduction ratios of hepcidin 20, 22, and 25 did not significantly differ between the PS and AN69 membranes [81]. 

The study thus demonstrated that the AN69 membrane had the potential to remove hepcidin because of its high adsorptive capacity, whereas PSu removed serum hepcidin because of its high solute-removing potential. In consideration of the high adsorptive capacity of the AN69 membrane for hepcidin, HD patients treated with AN69 membrane might need less quality of intravenous iron administration.

## 21. Production of Platelet-Activating Factor (PAF)

PAF is produced by different cells, such as macrophages, monocytes, platelets, neutrophils, and endothelial cells, via activation from immune or nonimmune stimuli. Apart from other biological functions, PAF mediates allergic responses. Interaction between blood cells and HD membranes stimulates PAF production. Therefore, the blood concentration of PAF is regarded as one of the important indices of membrane biocompatibility.

The production of PAF during HD with a CU membrane has been established; however, with the AN-69 PAN membrane, this has not been clearly linked. To assess this, a study [84] was conducted among 10 HD patients, who were subjected to HD with CU and AN-69 membranes for two consecutive weeks (first week with CU and second week with AN-69). The blood PAF levels and leukocyte and platelet counts were measured during the third HD session of each week and at different time points (0, 2, 5, 15, 30, 60, 180, and 240 min), while the circulating levels of the C3a-desArg and SC5b-9 were measured at 0, 5, 15, 60, and 240 min. The study results showed that circulating PAF levels were detectable at all time points during HD with AN-69 (PAF_AN-69_) and CU (PAF_CU_) membranes [83]. 

The study findings showed that at all time intervals PAF_AN-69_ < PAF_CU_, statistically significant differences (s) existed only at 15, 30, 60, 180, and 240 min between the two membranes. The highest PAF_AN-69_ and PAF_CU_ levels occurred at 5 and 15 min, respectively, during dialysis. Similar observations were made for the reduction in circulating leukocytes and C3a-desArg levels. The maximum reduction in platelet count was observed after two minutes of dialysis initiation for both membranes. The study concluded that although AN69 led to the production of PAF, circulating PAF levels were lower at all time intervals during HD with the AN 69 membrane when compared with the CU membrane. This study confirmed that PAF production with both the membranes probably contributed to thrombocytopenia and leukopenia. 

## 22. Effect on Patient Survival

A retrospective single-center study assessed the survival characteristics over a 10-year period of 340 HD patients who were hemodialyzed exclusively on PAN (polyacrylonitrile) or AN69 membranes and compared it with national data collected by the US Renal Data System (USRDS). The USRDS, established in 1989, is the largest national ESRD and CKD surveillance system in the United States. USRDS covers Medicare and non-Medicare ESRD patients, and Medicare CKD patients. USRDS is a stand-alone database on the diagnosis and demographic characteristics of ESRD patients, along with biochemical data, dialysis information, hospitalization, and deaths. The characteristics of 340 HD patients using PAN or AN69 membranes were: age of 55.89 ± 0.9 years (mean ± SE) and HD duration of 922.99 ± 47.58 days. The diagnostic categories were diabetic nephropathy (30%), glomerulonephritis (23.5%), hypertensive nephrosclerosis (18.5%), and others (28%). Corresponding information about age, mean duration of HD, and cause of renal failure in USRDS database were not provided in the manuscript [83]. The number of expected deaths in 340 patients according to the USRDS database was 190, whereas the number of the observed actual deaths in patients on PAN/AN69 membranes was only 120, a high significant difference at a *p* value of <0.0001 (Table 4) [83]. A comparison with the national data collected by the USRDS revealed that AN69 improved survival in HD membranes, possibly due to its better removal of ‘intermediate molecules’ and low-molecular-weight uremic toxins by AN69 [83].

## 23. Conclusions

As Japan has a relatively large number of dialysis patients, evaluating of the performance of dialysis membranes, in terms of biocompatibility, long-term benefits, and lowered dialysis-associated complications, is of paramount importance. The constantly increasing number of elderly patients on long-term dialysis in Japan is another issue of concern and therefore requires careful consideration of the appropriate dialysis membranes. 

The AN69 membrane has several advantages over other dialysis membranes. The key features of the AN69 membrane is its high permeability and selective absorptive property. The AN69 membrane improves nutritional status, lowers inflammation in patients undergoing dialysis, and leads to lower amino acid loss, implying a better nutritional state with its use in MHD patients. The AN69 membrane demonstrates good biocompatibility and HD efficiency in the setting of atherosclerosis and PAD in dialysis patients. Moreover, the safety profile of the AN69 membrane broadens its applicability and highlights its importance as the membrane of choice in chronic HD patients. 

## Figures and Tables

**Table 1:** Different types of dialysis membranes and their key features.

Biomaterial of Dialysis Membrane	Common Name	Key Features	
		Chemical Properties	Clinical Significance
**Cellulose**	Cuprophan (CU)	Composed of regenerated cellulose, consisting of linear chains of glucose rings with free surface hydroxyl groups Highly hydrophilic and uniform porosity	Low cost Good diffusive transport properties for small solutes
**DEAE-substituted cellulose**	Hemophan	Regenerated cellulose membrane with positively charged DEAE substance substituting 1% hydroxyl moieties	Higher suppression of complement activation Adsorbs heparin, causing blood coagulation
**Cellulose diacetate and triacetate**	CA, CTA	Modified cellulose membranes developed by substituting free hydroxyl groups on cellulose membrane surface with CA, or diacetate (80% substitution), or CTA (90% substitution) More hydrophobic than regenerated cellulose membranes	Lower complement activation vs. unsubstituted cellulose
**Multilayer vitamin-E coated cellulose**	Excebrane	Developed by covalent binding of synthetic block polymers (oleyl alcohol and vitamin-E moieties) to hydroxyl groups on cellulose Decreased activation of mononuclear cells and lower levels of proinflammatory cytokine, IL-6	Has antioxidant effects and reduces thrombosis Decreased activation of mononuclear cells and lower levels of proinflammatory cytokine, IL-6
**Polysulfone**	F-series	Synthetic polymeric membrane made from petroleum Hydrophobic in nature and mostly included with pore-forming hydrophilic agents	Relatively low diffusion rates for small solutes (to overcome this disadvantage, polyvinylpyrrolidone is added during the manufacturing process.)
	Optiflux series		
**Polyamide**	Polyflux series	Derived from synthetic polymer, polyamide Strongly asymmetric membrane structure	Due to hydrophobic sites, allows endotoxin retention Owing to minimal interaction with blood components, provides improved biocompatibility
**Polyacrylonitrile and methacrylate**	PAN	Synthetic membrane Hydrophobic	Adsorbs proteins and cells on their surface
**Poly(methyl methacrylate)**	PMMA	Synthetic membrane Hydrophobic	Adsorbs proteins and cells on their surface
**Polyethersulfone**	PES	Synthetic membrane Hydrophobic	Adsorbs proteins and cells on their surface
**Polycarbonate**	Polycarbonate	Synthetic membrane Hydrophobic	Causes activation of proteins and cells

(From [36,37,38,39,40], CU: Cuprophan; CA: Cellulose acetate or diacetate; CTA: Cellulose triacetate; DEAE: Diethylaminoethyl; PAN: Polyacrylonitrile; PMMA: Poly (methyl methacrylate); PES: Polyethersulfone.

**Table 2:** Membrane in chronic hemodialysis patients.

Author and Year	Study Type	Study Details	Dialysis Membranes Involved
**Furuta et al., 2011** [78]	A crossover study to compare HD efficiency and effects on nutritional, hemodynamic, and inflammatory conditions of polysulfone and AN69 membranes in elderly (aged 75 years or older) HD patients.	Twenty-eight elderly maintenance HD patients were treated with polysulfone for 3 months, followed by AN69 for the next 3 months, then switched back to polysulfone for 3 months.	Polysulfone and AN69
**Nakada et al., 2014** [79]	Crossover trial to study the efficacy of long-term use of PAN hemodialyzer in elderly dialysis patients with mild PAD.	Six chronic HD patients were switched from polysulfone to AN69 membrane and observed for 72 weeks.	AN69 and polysulfone membrane
**Yokomatsu et al., 2014** [80]	Comparative study to assess amino acid loss into dialysate during HD with 3 different membranes.	Nine maintenance HD patients were studied, who received HD for more than 3 months. Dialysate samples were evaluated for measurement of amino acid loss.	Nonhydrophilic PEPA (FLX-15GW, Nikkiso), hydrophilic PEPA (FDX-150GW, Nikkiso), PAN/ AN69 membrane (H12-4000, Gambro)
**Kuragano et al., 2013** [81]	Single-center study; measured changes in serum hepcidin levels during HD with different membranes.	Comprised ex vivo and in vivo studies. In the ex vivo study, a mini-dialyzer made of either polysulfone or AN69 was used to circulate blood from healthy volunteers, followed by measurement of serum hepcidin levels. In the in vivo study, 10 healthy individuals and 28 maintenance HD patients were included. After treatment with the polysulfone membrane, AN69 was used in the following weeks and serum hepcidin levels were measured.	APS-SA^®^ (hollow fiber, composed of polysulfone membrane), and H12-3400^®^ (flat-sheet, AN69)
**Latrou 2002** [82]	Comparative study to evaluate the effects of HD membranes on production of PAF.	The study was conducted among 10 HD patients who were first treated with the CU membrane and then the AN69 membrane in the following week. Along with changes in the PAF levels at different time points, platelet and leukocyte counts and the extent of complement activation were studied.	CU and AN69 membranes
**Chandran 1993** [83]	A ten-year analysis to study patient survival on PAN/AN69 HD.	This was a retrospective single-center study conducted to analyze the 10-year survival of 352 HD patients on PAN/AN69 membrane.	PAN/AN69 membrane

(From [78,79,80,81,82,83], MIA: malnutrition, inflammation, and atherosclerosis; HD: hemodialysis; AN69-ST: AN69 surface-treated; ESRD: end-stage renal disease; PAN: polyacrylonitrile; PAD: peripheral arterial disease; PEPA: polyester–polymer alloy; PAF: platelet-activating factor; CU: cuprophan.

**Table 3:** Clinical Evidence on Benefits of AN69 in Inflammation, Malnutrition, and Peripheral Arterial Disease Associated with Hemodialysis.

Amino Acids	Hydrophilic PEPA	Non-Hydrophilic PEPA	AN69	*p*-Value
**Ornithine**	2.0 ± 0.6	2.0 ± 0.4	1.4 ± 0.4	0.008 *
**Phenylalanine**	2.4 ± 0.9	2.3 ± 0.5	1.8 ± 0.8	0.005 **
**Tryptophan**	0.6 ± 0.2	0.7 ± 0.2	0.4 ± 0.2	0.002 **
**Cystine**	2.8 ± 1.4	3.2 ± 0.7	2.0 ± 0.7	0.004 **

PEPA: Polyester–polymer alloy; * *p* ≤ 0.05; ** *p* ≤ 0.005.

**Table 4:** Survival of hemodialysis patients treated with PAN/AN69 membrane [83].

No. of Patients on PAN/AN69 in Authors’ HD Center	Age (Mean ± SE)	HD Duration (Days)	Expected Mortality from USRDS Database	Observed Mortality	*p*-Value
340	55.89 ± 0.9	922.99 ± 47.58	189.89	120	<0.001

PAN: Polyacrylonitrile; SE: Standard error.

## Data Availability

There is no data to provide for reasonable request in this review article.
